# The Application of and Factors Influencing, the NB5 Assay in Neuroblastomas

**DOI:** 10.3389/fonc.2021.633106

**Published:** 2021-05-14

**Authors:** Zuopeng Wang, Chengyun Wang, Yibing Xu, Jun Le, Yuan Jiang, Wei Yao, Hongsheng Wang, Kai Li

**Affiliations:** ^1^ Department of Pediatric Surgery, Children’s Hospital of Fudan University, Shanghai, China; ^2^ Department of Pediatric Surgery, Zaozhuang Maternal and Child Health Care Hospital, Shandong, China; ^3^ Institute of Translational Medicine, Zhejiang University School of Medicine, Hangzhou, China; ^4^ Department of Hematology, Children’s Hospital of Fudan University, Shanghai, China; ^5^ Department of Clinical Epidemiology, Children’s Hospital of Fudan University, Shanghai, China

**Keywords:** neuroblastoma, NB5 assay, sensitivity, micrometastases, bone marrow

## Abstract

**Purpose:**

The NB5 assay was performed in bone marrow (BM) and peripheral blood (PB) to detect neuroblastomas (NBs) with micrometastases. The sensitivity and factors influencing the NB5 assay were preliminarily evaluated.

**Methods:**

The NB5 assay uses RT-PCR to detect the co-expression of five mRNAs from the neuroblastoma-associated genes, CHGA, DCX, DDC, PHOX2B, and TH. We enrolled 180 cases of neuroblastoma and 65 cases of non-neuroblastoma. Bone marrow and peripheral blood were collected from every patient. The gold standard for the diagnosis of NB was pathological evaluation of solid tumor specimens or bone marrow biopsies (BMBs) from hematological tumors. STATA version 15 and SPSS version 17 software were used for analysis.

**Results:**

We found that 17 patients were BMB (+), and they were diagnosed as the International Neuroblastoma Staging System (INSS) stage IV and the high-risk group. All 17 patients were BM (+), while 15 patients were PB (+) (15/17, 88.2%). Among the 163 children who were BMB (−), 56 were BM (+), 40 were PB (+), and 36 were BM (+) and PB (+). The sensitivity of the NB5 assay in BM (40.5%) and PB (30.5%) was significantly higher than the sensitivity of BMB (9.4%, P = 0.000). In the non-NB group, four cases were BM (+) and one case was PB (+). The specificity of the NB5 assay in BM and PB was 93.8% and 98.5%, respectively. The sensitivity of the NB5 assay in both BM and PB in INSS stage IV patients was significantly higher than that in INSS stage I–II patients (P <0.05). The sensitivity of the NB5 assay in both BM and PB in the high-risk group was significantly higher than that in the middle-low-risk groups (P = 0.0001). Logistic regression analyses indicated that liver metastases and bone metastases were the primary factors influencing the sensitivity of the NB5 assay in BM and PB (P <0.05).

**Conclusions:**

The NB5 assay had significantly higher sensitivity than the pathological analysis of BMB in detecting NB with micrometastases. The NB5 assay had higher sensitivity in INSS stage IV or the high-risk group. Liver metastases and bone metastases were the primary factors that affected the sensitivity of the NB5 assay.

## Introduction

Neuroblastoma (NB) is the most common extracranial solid tumor in children and accounts for 15% of all pediatric malignancy deaths (1). Although NB has a heterogeneous clinical course and may regress spontaneously, most patients with NB experience early onset and progress rapidly. Approximately 45% of patients have distant metastatic lesions when diagnosed during infancy (2). NB with micrometastases are known as the minimal residual disease (MRD) and contribute to relapse, but are difficult to detect (3). The persistence of MRD is also predictive of worse patient survival and poorer outcomes (4). Bone marrow biopsies (BMBs) are routinely used for the diagnosis of bone marrow (BM) metastasis by cytological and histological examinations, which exhibit an analytical sensitivity of less than 1 × 10^−3^. Thus, such sensitivities could severely underestimate the prevalence of bone marrow involvement (1, 2, 5, 6). Immunocytological investigations or flow cytometry had an analytical sensitivity of up to (1 × 10^−4^) − (1 × 10^−5^) for MRD in NB (5). With well-defined reverse transcriptase polymerase chain reaction (RT-PCR) markers that could provide a sensitivity of MRD of 1 × 10^−6^, one NB cell among 10^6^ normal cells could be detected for the early diagnosis of NB.

Several MRD diagnostic methods based on the expression of multiple gene markers have been reported (7–11). As a novel diagnostic method for NB, the mRNAs of five neuroblastoma markers (NB5 assay), including chromogranin A (CHGA), doublecortin (DCX), dopadecarboxylase (DDC), paired-like homeobox 2b (PHOX2B), and tyrosine hydroxylase (TH) were detected in the BM and peripheral blood (PB) from patients using RT-PCR (8). The NB5 assay was developed at the Children’s Hospital of Los Angeles and is very sensitive in detecting MRD in BM and PB. Shanghai Bokang Biotechnology Co. LTD performed the detection services for MRD in NB using the NB5 assay.

The current consensus is that MRD remains in a dormant state, until “awakened” to progress towards overt metastases (12). MRD existing in the blood and bone marrow could contain tumor-initiating cells that generate tumors through abnormal proliferation and differentiation (13). A good MRD detection marker should be exclusively expressed in NB cells and not in non-NB cells; however, no clinical studies evaluated the sensitivity and effectiveness of these methods with NB markers. In this study, the NB5 assay was performed to assist in the clinical diagnosis of NB, and its sensitivity and specificity were preliminarily evaluated.

## Methods

### Patients

Patients were regarded as having micrometastases when the expression of three or more genes was detected. Between August 2015 and May 2019, we enrolled 180 NB cases and 65 non-NB cases from the Children’s Hospital of Fudan University. This study received approval from the local Research Ethics Committee of our hospital and was performed in accordance with the approved guidelines. Written informed consent was obtained from the guardians of each patient. The gold standard for the diagnosis of NB was based on pathological evaluations of solid tumor specimens or the BMBs of hematologic tumors.

### Sample Processing and NB5 Assay

BM (2 ml) and PB (2 ml) were collected from each patient with a solid tumor or hematologic tumor, respectively. Peripheral blood mononuclear cells (PBMCs) were isolated from heparinized blood and bone marrow by density separation with Ficoll–Hypaque (14). Total RNA was prepared using the TRIzol^®^ reagent (Invitrogen) and processed with the RNeasy^®^ Mini Kit (QIAGEN). The RNA Integrity Number (RIN) was obtained using the Agilent Bioanalyzer, and only specimens with RIN >5.5 were tested. Reverse transcription of 2,500 ng of total RNA in 20 μl was carried out with M-MLV Reverse Transcriptase (Life technologies). The NB5 assay quantified the expression of the neuroblastoma-associated genes, CHGA, DCX, DDC, PHOX2B, and TH, as well as the housekeeping gene, beta-2-microglobulin (B2M). Predesigned and preoptimized probes and primer sets (**Supplementary Table S1**) were included with 2,500 ng of cDNA and amplified using standard cycling conditions using the 7900HT Fast Real-Time PCR System (Applied Biosystems). The ΔCt was chosen instead of Ct alone as ΔCt accounts for the RNA quality of the samples obtained. Note that ΔCt values may be equivalent when one specimen has no detectable NB mRNA, and other specimen is mildly positive. The detection service of NB5 assay was supplied by Shanghai Bokang Biotechnology Co. LTD.

### Statistical Methods and Software

The sensitivity and specificity of the NB5 assay were determined using STATA version 15 software. The chi-square test and logistic regression analyses were carried out using SPSS version 17 software. P-values <0.05 were considered statistically significant.

## Results

### Patient Demographics

The NB group included 101 males and 79 females aged from 1 month to 10 years. Patient characteristics, International Neuroblastoma Staging System (INSS) stage, risk group, and clinical data are listed in **Table 1**. The non-NB group contained 65 diverse hematological tumors and solid tumors, including nephroblastoma (n = 12), pancreatoblastoma (n = 4), retinoblastoma (n = 4), pheochromocytoma (n = 1), adrenocortical carcinoma (n = 1), rhabdomyosarcoma (n = 5), teratoma (n = 5), lymphoma (n = 10), lymphocytic leukemia (n = 16), hepatoblastoma (n = 5), primitive neuroectodermal tumor (n = 1), and an endodermal sinus tumor (n = 1). The clinical features of metastasis in the non-NB group are listed in supplementary **Table S2**.

**Table 1:** Clinical characteristics of NB patients.

Total cases	N	(%)
Age (mo)		
<12	43	23.89%
≥12	137	76.11%
Sex		
Female	79	43.89%
Male	101	56.11%
Primary site		
Abdomen	162	90.00%
Thorax and other	18	10.00%
Tumor size		
>10 cm	65	36.11%
≤10 cm	115	63.89%
Tumor stage		
I	37	20.56%
II	17	9.44%
III	21	11.67%
IV	89	49.44%
Ivs	16	8.89%
Risk group		
Low risk group	97	53.89%
medium risk group	42	23.33%
high-risk group	41	22.78%
MYCN gene		
Amplification	27	15.00%
Nonamplification	153	85.00%
NSE (ng/mL)		
<370	131	72.78%
≥370	49	27.22%
Metastatic site		
Bone	35	19.44%
Bone marrow	17	9.44%
Lymph node	97	53.89%
Liver	23	12.78%

### The Sensitivity and Specificity of the NB5 Assay

We identified NB cells in 17 patients based on the evaluation of BMBs. All such individuals were clinically diagnosed as the INSS stage 4, high-risk group. All 17 patients were BM (+), while 15 patients were PB (+) (15/17, 88.2%). Among the 163 children who were BMB (–), 56 were BM (+), 40 were PB (+), and 36 were BM (+) and PB (+). The sensitivity of the NB5 assay in BM (40.6%) was significantly higher than that of BMBs (9.4%, P = 0.000). Similarly, the sensitivity of the NB5 assay in PB (30.5%) was significantly higher than that of BMBs (9.4%, P = 0.000). However, there was no significant difference between the sensitivity of the NB5 assay in BM and PB (P = 0.103).

In the non-NB group, four cases were BM (+), including those with retinoblastoma (n = 2), nephroblastoma (n = 1), and teratoma (n = 1). One case was PB (+), which was an endodermal sinus tumor (n = 1).

The sensitivity and specificity of the NB5 assay in BM were 40.5% (95%CI, 33.3–48.1%) and 93.8% (95%CI, 85–98.3%), respectively. The likelihood ratio (−), the likelihood ratio (+), the negative predictive value, and the positive predictive value are shown in **Table 2**.

**Table 2:** Sensitivity and specificity of the NB5 assay in BM.

			[95% Confidence Interval]
Sensitivity	Pr(+|A)	40.60%	33.30–48.10%
Specificity	Pr(−|N)	93.80%	85–98.30%
ROC area	(Sens. + Spec.)/2	0.672	0.626–0.718
Likelihood ratio (+)	Pr(+|A)/Pr(+|N)	6.59	2.51–17.3
Likelihood ratio (-)	Pr(-|A)/Pr(−|N)	0.633	0.553–0.726
Odds ratio	LR(+)/LR(−)	10.4	3.77–28.6
Positive predictive value	Pr(A|+)	94.80%	87.20–98.60%
Negative predictive value	Pr(N|−)	36.30%	29–44.10%

**Figure 1:**
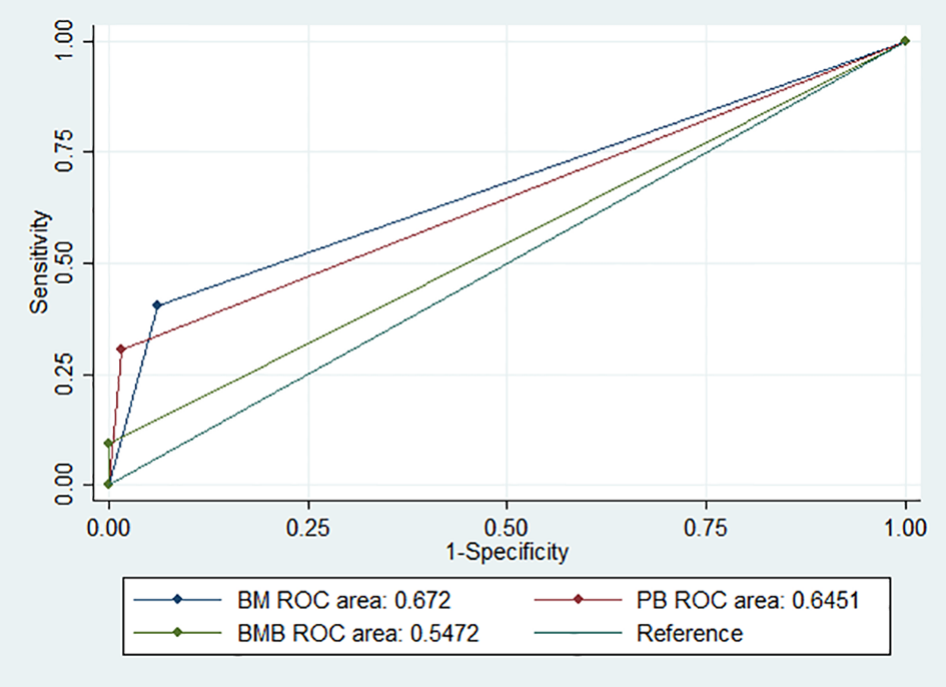
ROC curves indicating the sensitivity of the NB5 assay in BM [area, 0.672 (95% CI 0.625–0.718)]; PB [area, 0.645 (95% CI 0.608–0.682)]; and BMB [area, 0.547 (95% CI 0.525–0.568)].

The sensitivity and specificity of the NB5 assay in BM were 30.6% (95%CI, 23.9–37.8%) and 98.5% (95%CI, 92–100%), respectively. The likelihood ratio (−), the likelihood ratio (+), the negative predictive value, and the positive predictive value are shown in **Table 3**. The receiver operating characteristic (ROC) curves of the sensitivity of the NB5 assay and BMB indicated that the NB5 assay in PB and BM samples exhibited significantly higher sensitivity than BMB (**Figure 1**).

**Table 3:** Sensitivity and specificity of the NB5 assay in PB.

			[95% Confidence Interval]
Sensitivity	Pr(+|A)	30.60%	23.90–37.80%
Specificity	Pr(−|N)	98.50%	92–100.00%
ROC area	(Sens. + Spec.)/2	0.645	0.608–0.682
Likelihood ratio (+)	Pr(+|A)/Pr(+|N)	19.9	2.81–141
Likelihood ratio (-)	Pr(−|A)/Pr(−|N)	0.705	0.637–0.781
Odds ratio	LR(+)/LR(−)	28.2	4.8–0
Positive predictive value	Pr(A|+)	98.20%	90.40–100.00%
Negative predictive value	Pr(N|−)	33.90%	27–41.10%

Pr, proportion; Sens, sensitivity; Spec, specificity; LR, likelihood ratio; +, positive; −, negative; A, Abnormal; N, Normal.

### Analyses of the Sensitivity of the NB5 Assay

The sensitivity of the NB5 assay in BM and PB was compared to BMBs from different INSS stages (**Table 4**). The results revealed significant differences between the NB5 assay and BMBs in INSS stage III and IV samples. The sensitivity of the NB5 assay in BM and PB from INSS stage IV samples was significantly higher than that in INSS stage I–II samples (P < 0.05). The sensitivity of the NB5 assay in BM and PB in samples from the high-risk group was significantly higher than that in samples from the middle-low-risk groups (P = 0.000). The sensitivity of the NB5 assay between samples from different INSS stages are shown in **Table 5**.

**Table 4:** Sensitivity of the NB5 Assay between BM, PB, and BMBs from different INSS stages.

	N	BM	BMB	p value	
				BM *vs* BMB	PB *vs* BMB
I	37	4	0	0.071	0.135
II	17	2	0	0.271	0.514
III	21	7	0	**0.014**	**0.024**
IV	89	55	17	**0.0001**	**0.004**
IVs	16	5	0	**0.047**	0.082
Total	180	73	17	**0.0001**	**0.0001**

Bold value means p < 0.05 which showed significant difference.

**Table 5:** Sensitivity of the NB5 assay in BM and PB from different INSS stages.

	BM				PB			
	II	III	IV	IVs	II	III	IV	IVs
I	0.626	0.087	**0.00001**	0.135	0.635	0.087	**0.01**	0.159
II		0.197	**0.012**	0.248		0.137	**0.015**	0.205
III			0.131	0.597			0.236	0.574
IV				0.149				0.219

Bold value means p < 0.05 which showed significant difference.

### The Factors Influencing the Analysis of the NB5 Assay

We carried out logistic regression analyses of liver metastases, bone metastases, lymph node metastases, tumor size >10 cm, neuron-specific enolase (NSE) ≥370 ng/ml, and MYCN with the NB5 assay. The results revealed that liver metastases (P = 0.028) and bone metastases (P = 0.002) affected the sensitivity of the NB5 assay in BM. Three factors had significant differences in the NB5 assay in PB, including liver metastases (P = 0.0001), bone metastases (P = 0.018), and NSE (P = 0.035). No other factors were significantly different (P > 0.05) (**Table 6**).

**Table 6:** Factors influencing the analysis of the BN5 assay in BM and PB.

P value	Liver	Lymph node	Bone	Tumor size>10 cm	NSE>370	MYCN
BM	**0.028**	0.332	**0.002**	0.391	0.261	0.069
PB	**0.018**	0.445	**0.0001**	0.153	**0.035**	0.284

Bold value means p < 0.05 which showed significant difference.

## Discussion

Previously, CHGA, DCX, DDC, PHOX2B, and TH were suggested as indicators of micrometastases in NB by single mRNA or combinations (9, 11). Several studies suggested that the analysis of multiple genes was more informative than the analysis of a single gene in detecting NB cells (15–18). In fact, the combination of all five genes is more sensitive in detecting NB micrometastases (8).

PHOX2B encodes a homeodomain transcription factor involved in the differentiation and development of several major noradrenergic neural cells (19). PHOX2B is highly expressed in NB and has been reported as a specific marker for MRD in NB (17, 20). DCX is specifically expressed in migrating neurons of the central and peripheral nervous systems, and regulates the microtubule cytoskeleton by signaling pathway (21). CHGA is a neuroendocrine marker that participates in coding for neurosecretory granules that promote the differentiation of NB cells (22). TH encodes the first enzyme involved in the catecholamine synthesis pathway, which serves a functional role in the detection of NB with micrometastases, as catecholamines are mainly produced by NB cells (3). Similar to TH, DDC is a key enzyme involved in catecholamine synthesis and has been claimed to be a sensitive marker for NB (23).

The present study focused on assessing the sensitivity and specificity of the NB5 assay in clinical applications (8) and explored the factors influencing the assay. The sensitivity and specificity of the assay in BM were 40.5% and 93.8%, while they were 30.6% and 98.5%, respectively, for PB samples. We found that the sensitivity of the NB5 assay in BM and PB was significantly higher than that of BMB; however, there was no significant difference between the assay sensitivity in BM and PB (P = 0.103). The sensitivity of the NB5 assay in BM and PB samples from INSS stage IV samples was significantly higher than that in INSS stages I–II samples, while the sensitivity in BM and PB in the high-risk group was significantly higher than that in the middle-low-risk groups. Logistic regression analyses indicated that liver metastases and bone metastases were the primary factors influencing the sensitivity of the NB5 assay in BM and PB. Liver and bone metastases are transferred through the blood, and thus, there is a significant correlation with the detection efficiency of the NB5 assay. Lymph nodes are transferred through the lymphatic system, and therefore, have little impact on the NB5 assay in BM and PB. The NB5 assay results were not associated with tumor size, but may be associated with tumor biology. NSE >370 ng/ml was the factor influencing the NB5 assay in PB (P = 0.035), while MYCN was a possible influencing factor in BM (P = 0.069).

Except as diagnostic methods, Virginie et al. suggested that the mRNA levels of PHOX2B, TH, and DCX in BM could be used as predictors of event-free survival (EFS) and overall survival (OS) and also to monitor the statuses of patients throughout their course of treatment for NB (16). Janine et al. found that the expression of these mRNAs varied greatly during the treatment of NB or during relapse, which rendered them excellent markers (24). However, Alexander et al. found that flow cytometric analysis for NB cells in BM was much stronger than mRNA detection in prognostic impact (25). Thus, based on this study, the prediction of the NB5 assay in the relapse and prognosis of NB warranted further evaluation, especially compared with flow cytometric analyses.

Illegitimate expressions were investigated in the non-NB group, including in retinoblastoma (n = 2), nephroblastoma (n = 1), and teratoma samples (n = 1) in BM (+) cases, and in an endodermal sinus tumor sample (n = 1) in a PB (+) case. However, most of such samples were weakly positive in the NB5 assay. Among the five genes evaluated for mRNA expression, PHOX2B was a more specific biomarker for NB than TH, DDC, CHGA, or DCX. The mRNA expression of TH, DDC and DCX was observed in hematopoietic cells and PB from healthy donors, while PHOX2B expression was limited to only NB samples (7, 9, 26). Despite the small number of false positives and false negatives in the NB5 assay, we will now conduct a larger multicenter study, combined with clinical characteristics and multiple other tests for further verification of NB. To maximize the sensitivity and specificity of the NB5 assay, we will optimize the assay to improve its accuracy of diagnosis.

## Conclusion

In summary, the NB5 assay had significantly higher sensitivity in detecting NB with micrometastases in BM and PB than BMB. The NB5 assay had higher sensitivity in patients with INSS stage IV or in the high-risk group. Liver metastases and bone metastases were the factors that affected the sensitivity of the NB5 assay in BM and PB samples. In the future, we will analyze the relationship between the NB5 assay and prognosis and explore the relationship between tumor relapse and PHOX2B, TH, DDC, CHGA, and DCX expression.

## Data Availability Statement

The raw data supporting the conclusions of this article will be made available by the authors, without undue reservation.

## Ethics Statement

The studies involving human participants were reviewed and approved by the local Research Ethics Committee of Children’s Hospital of Fudan University. Written informed consent to participate in this study was provided by the participants’ legal guardian/next of kin.

## Author Contributions

KL, HW and ZW conceived and designed the study. CW, ZW, JL and YX collected the clinical data, performed data analysis, and wrote the paper. YJ and WY offered the assist in data collection. KL, ZW, HW and YX reviewed and edited the manuscript. All authors contributed to the article and approved the submitted version.

## Funding

This study was sponsored by the Personnel Training Program for Distinguished Medical Young Scholar (2017, to KL), the New Hundred People Plan of Shanghai Health and Family Planning Commission (2017 BR052, to KL), the Shanghai Sailing Program (17YF1401400, to ZW), the Youth Fund of Shanghai Health and Family Planning Commission Clinical Research Special Plan (20184Y0212, to ZW), the Clinical Research Plan of SHDC (no. SHDC2020CR2009A) and the Shanghai Municipal Key Clinical Specialty (no. shslczdzk05703).

## Conflict of Interest

The authors declare that the research was conducted in the absence of any commercial or financial relationships that could be construed as a potential conflict of interest.
