# Supplementary material for: The Application of and Factors Influencing, the NB5 Assay in Neuroblastomas
Source: Front Oncol. 2021 May 14;11:633106. doi: 10.3389/fonc.2021.633106 (PMC8162211; doi:10.3389/fonc.2021.633106)
Supplement: Supplementary file 1 [file Table_1.docx]

**Table S1. Genes, and primer and probe sets for the NB5 assay**

| Gene | Primer and Probe Set |
| --- | --- |
| Neuroblastoma |  |
| CHGA | CHGA-Hs00154441_m1 |
| DCX | DCX-Hs00167057_m1 |
| DDC | DDC-Hs00168031_m1 |
| PHOX2B | PHOX2B-Hs00243679_m1 |
| TH | TH-Hs00165941_m1 |
| Housekeeping |  |
| B2M | B2M-Hs99999907_m1 |
