# Supplementary material for: The Application of and Factors Influencing, the NB5 Assay in Neuroblastomas
Source: Front Oncol. 2021 May 14;11:633106. doi: 10.3389/fonc.2021.633106 (PMC8162211; doi:10.3389/fonc.2021.633106)
Supplement: Supplementary file 2 [file Table_2.docx]

**Table S2.** The clinical features of metastasis in the non-NB group

| Non-Neuroblastoma | Number |
| --- | --- |
| Solid tumor | 39 |
| Nephroblastoma | 12 |
| Metastasis | 4 |
| No metastasis | 8 |
| Rhabdomyosarcoma | 5 |
| Metastasis | 1 |
| No metastasis | 4 |
| Teratoma | 5 |
| Metastasis | 2 |
| No metastasis | 3 |
| Hepatoblastoma | 5 |
| Metastasis | 0 |
| No metastasis | 5 |
| Pancreatoblastoma | 4 |
| Metastasis | 1 |
| No metastasis | 3 |
| Retinoblastoma | 4 |
| Metastasis | 2 |
| No metastasis | 2 |
| Pheochromocytoma | 1 |
| Metastasis | 1 |
| No metastasis | 0 |
| Adrenocortical carcinoma | 1 |
| Metastasis | 1 |
| No metastasis | 0 |
| Primitive neuroectodermal tumor | 1 |
| Metastasis | 1 |
| No metastasis | 0 |
| Endodermal sinus tumor | 1 |
| Metastasis | 0 |
| No metastasis | 1 |
| Hematologic malignancies | 26 |
| Lymphocytic leukemia | 16 |
| Low risk | 9 |
| Median risk | 3 |
| High risk | 4 |
| Lymphoma | 10 |
| IV | 5 |
| III | 5 |
| II | 0 |
| I | 0 |
